# A brief literature review of low-level laser therapy for treating amyotrophic lateral sclerosis and confirmation of its effectiveness

**DOI:** 10.37796/2211-8039.1430

**Published:** 2024-03-01

**Authors:** Sergey V. Moskvin

**Affiliations:** Scientific and Research Centre Matrix, Moscow, Russia

**Keywords:** Low-level laser therapy, Amyotrophic lateral sclerosis

## Abstract

**Introduction:**

Amyotrophic lateral sclerosis (ALS) is a neurodegenerative disease with a steadily progressive course due to the death of central and peripheral motor neurons responsible for voluntary movements. Low-level laser therapy (LLLT) is a treatment method unique in its universality and efficacy, particularly for neurodegenerative diseases.

**Methods:**

In this review, we discuss the effect and application of LLLT in the treatment of ALS. A literature search for English and Russian publications for the keywords “Amyotrophic Lateral Sclerosis”, “Low-Level Laser Therapy” was performed using PubMed, Scopus, Google Scholar, Web of Science and Russian Science Citation Index (RSCI) databases.

**Results:**

The article provided a brief literature review, substantiated the potential use of low-level laser therapy for ALS. The particular techniques of LLLT were developed.

**Conclusion:**

Based on the results of several studies and many years of successful experience with low-level laser therapy in Russia we conclude that a LLLT technique, including intravenous laser blood illumination (ILBI), noninvasive laser blood illumination (NLBI), and local exposure, is a promising treatment method for ALS.

## 1. Introduction

A myotrophic lateral sclerosis (ALS) is a neurodegenerative disease with a steadily progressive course caused by the death of central and peripheral motor neurons responsible for voluntary movements [1]. ALS is a multifactorial disease in which both exogenous effects and genetic predisposition play a role. The main external pathogenic factors are chronic occupational head injuries (athletes, military, etc.), exposure to various toxic substances (metals, pesticides, insecticides, and organic solvents), and smoking. A role for exposure to electromagnetic fields in ALS has not been proven [2,3].

The multifactorial nature of the disease explains the current lack of specific etiotropic therapies for ALS, as it involves a whole cascade of pathological reactions, ultimately leading to motor neuron death. These include excitotoxicity, oxidative stress, mitochondrial dysfunction, conformational changes of proteins and protein aggregation, proteolytic system imbalance, disruption of cytoskeleton proteins and axonal transport, RNA processing and calcium homeostasis, deficiency of neurotrophic factors, and microglia activation [2,4–6].

The annual incidence of ALS worldwide averages about two (0.2–2.4) cases per 100,000 people, with a prevalence of about 5 (0.8–7.3) cases per 100,000 people. The ratio of males to females with classical ALS is 1.5:1. However, this ratio may differ, according to the various clinical forms of the disease. The age of disease onset is from 20 to 80 years, but onset is most common between the ages of 50 and 65 years. The average survival time after a diagnosis of ALS is 30 months, although survival times of up to 10 years have been recorded in those with a mild disease course. According to various epidemiological studies, the annual incidence of ALS in Russia is 2.5–2.9 per 100,000 people, with up to 8000 ALS patients as of 2020 [1,2].

At the same time, effective treatment methods have not yet been developed. Recommendations suggest drugs and methods that control only the main symptoms and correct respiratory disorders and nutritional deficiencies.

This study aims to analyze the potential of low-level laser therapy for treating ALS patients from the current understanding of the mechanisms of bio-modulating action of low-intensity laser illumination (BA LILI) and to propose effective techniques and recommendations to optimize their parameters.

## 2. Literature review

Conservative management of ALS includes drug treatment and methods aimed at controlling the main symptoms of the disease, such as respiratory disorders and nutritional insufficiency. Other common symptoms are muscle weakness, dysphagia, shortness of breath, pain, weight loss, speech disorders, constipation, coughing, sleep disturbances, emotional lability, and excessive salivation [1,4,7].

**Fig. 1:**
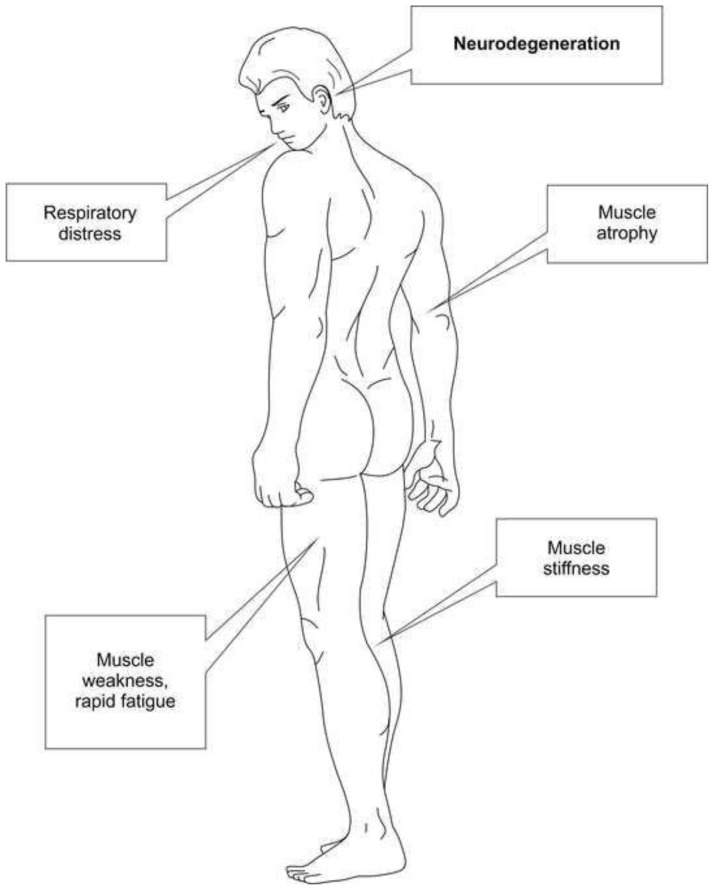
Affected areas in ALS.

Fig. 1 shows localization of the main disorders. Comparison of the pathogenesis of ALS with the mechanisms of BA LILI and the results of experimental and clinical studies allows many specialists to consider low-level laser therapy a promising treatment method [5,7–15].

ALS is generally considered in the context of neurodegenerative diseases, such as Parkinson's and Alzheimer's. Despite differences in the clinical picture of ALS versus that of other neurodegenerative diseases and differences in the type and localization of affected neurons, there are many similarities in terms of the mechanisms of nerve tissue destruction.

A common feature of all publications on the use of LLLT for ALS treatment, without exception, is the lack of recommendations on specific treatment techniques. We believe the above is primarily due to the indiscriminate choice of light sources, mixing lasers, LEDs, and bulbs. Ignoring monochromaticity, the most important factor determining the effectiveness of the therapeutic effect of electromagnetic radiation of the optical range, leads to a dead end, which together with a lack of understanding of the primary mechanism of BA LILI, means it is not possible, even in general terms, to understand where, what, and how to illuminate.

Russian researches were the first to report the use of low-level laser therapy (LLLT) for neurodegenerative diseases and to demonstrate high effectiveness of this method [16–19].

It is logical to choose intravenous laser blood illumination (ILBI) as a *systemic* method of exposure to address *systemic* health problems caused by neurodegenerative processes, which, in turn, are the consequence of pathological changes at the level of the body as a whole. As we have recently demonstrated, many systemic disorders are nonspecific and the result of the failure of complex neuro-immune-endocrine-metabolic interactions, which are an integrated whole, making it impossible to isolate any one component “responsible” for the system disruption [20].

LLLT acts as a universal regulator capable of restoring regulatory processes at all levels, from the cellular level to the entire organism, eliminating the basis for developing autoimmune, metabolic, neurodegenerative, and other diseases.

The aforementioned literature and other recently published studies provide a fairly comprehensive picture of the positive action of LLLT:

– Improving the interaction between astrocytes and neurons [21,22];
– Protecting motor neurons from the negative effects of reactive oxygen forms and neurotoxicity caused by oxygen-glucose deprivation through inhibition of neuronal nitric oxide synthase (nNOS) activity [23,24];
– Stimulating ATP synthesis and energy metabolism [25–27];
– Preventing oxidative stress and reduction of inflammation [26,28];
– Reducing muscle fatigue [27,29];
– Increasing muscle performance [30,31];
– Reducing muscle soreness after physical activities [25,32,33];
– Eliminating distress syndrome and restoring normal breathing [34].

The literature on the use of LLLT encompasses many more studies than those cited above.

The presence of opposite changes in autophagy proteins and phosphorylation of mTOR proteins (LC3-II, Beclin 1, p62/SQSTM1) in muscle cells and motor neurons, revealed in spinal muscular atrophy [35] demonstrates the specificity of the tissue regulatory system. On the other hand, it raises the question about the use of treatment methods that can simultaneously and comprehensively (i.e., systemically), influence the mechanisms of physiological regulation.

A recent study conducted by researchers in the USA showed that LLLT was significantly more effective in treating older patients than younger patients to enhance time to task failure, prevent loss of muscular strength and delay the onset of musculoskeletal fatigue. This shows increased sensitivity of older patients to laser light [36]. Russian researchers are well aware of the increased effectiveness of LLLT in the older population and have, for at least the last 40 years, recommended decreasing the power and/or exposure when treating children and the elderly with laser illumination [37].

A meta-analysis of experimental and preclinical studies showed an 11.2 % increase in survival in the treated mice after antioxidant therapy and a significant improvement (59.6 %) in muscle function. Methods of combatting oxidative stress include LLLT [38].

Analyzing the possibilities of laser therapy, R. Bedlack et al. (2022) [8] emphasized three preclinical studies that, in their opinion, reinforce the prospects of the method.

In the first study, using continuous infrared LILI (λ = 810 nm, power density [PD] 25 mW/cm^2^, 2-min exposure), the authors illuminated cultured mouse cortex neurons exposed to various excitotoxins. Its findings indicated that mitochondrial membrane potential and rate of ATP synthesis increased and the previously increased intracellular Ca^2+^ ion concentration and oxidative stress decreased, thus preventing cell death [39].

Another study, which is likely to be the only one that specifically studied ALS, compared clinical and histological features in G93A mSOD1 mice. The mice were divided into four groups: the control group (n = 12) received no treatment, the second group (n = 11) were fed a diet supplemented with riboflavin, the third group (n = 11) received percutaneous laser illumination (LI) (λ = 810 nm, continuous mode, power 140 mW, PD 100 mW/cm^2^, 2-min exposure) at three sites daily and the fourth group (n = 11) received a combination of riboflavin and LI. The treatment resulted in decreased glial fibrillary astrocyte protein staining, improved body weight, and temporary preservation of motor activity (until Day 130) without changing the number of motor neurons [40].

The authors of this study associated the lack of the LILI effect on the survival rate with the improperly chosen parameters of the LI technique. As for motor activity, they observed a short-term but significant improvement in the rotating rod test in the group receiving only LI. In other words, LLLT delayed the onset of movement disorders. Presumably, LI cannot inhibit the pathological processes of mitochondrial vacuolization and degeneration, which disrupt the electron transfer chain. This explains the reduced efficacy of LLLT in the later stages of the disease. In support of this theory, H. Moges et al. (2009) [40]carried out a series of in-vitro experiments using neuronal cells with an improperly assembled electron transport chain, demonstrating that the integrity of the mitochondrial electron transport chain is crucial for the effect of LLLT on cellular respiration and axonal transport [41]41.

The third publication reviewed clinical outcomes in 20 dogs with canine degenerative myelopathy (a natural disease caused by mutations in the SOD1 canine form) who received two different variants of percutaneous LLLT combined with physical therapy. These are group A (λ = 904 nm, power 0.5 W, PD 0.5 W/cm^2^, 20 s per point, 5 min total, 1–2 times per week) and group B (λ = 980 nm, power 6–12 W, PD 1.2–2.4W/cm^2^, continuous scan at 2.5–7.5 cm/s, 25–26 min total per treatment, 1–2 times per week). Dogs from group B retained the ability to walk longer and showed better survival than group A that hadless intensive exposure and stable technique [42]. It is impossible to evaluate this work from the point of view of LLLT technique parameters; the authors’ description of treatment is incorrect based on the instructions for the used devices.

R. Bedlack et al. (2022) [8] also found a brief report of one case published in the conference proceedings. It described a 69-year-old man with 4 years of progressive ALS with a diagnosis confirmed by neurological examinations, electromyography, and neuroimaging. At the beginning of LLLT, he was in a wheelchair and used a BiPAP (biphasic positive airway pressure) machine at night.

LLLT involved 2 modes: 1) λ = 810 nm, continuous mode, PD 30 mW/cm^2^, spot area 5 cm^2^ and 2) λ = 890 nm, pulsed mode, PD 10 W/cm^2^, frequency 250 Hz, light spot area 1 cm^2^. Illumination was performed percutaneously on the forehead, spine, brachial plexus, sternum, umbilicus, and pelvis, at 3 cycles with a 40-day break between them, each consisting of 2 treatments per day for 10 days. As reported, LLLT improved breathing and limb strength, and the patient regained the ability to hold a knife and fork, button a shirt, and walk more than 100 m. These benefits were transient, with regression noted 20–30 days after each cycle [43].However, this work contains insufficient information about the LLLT technique; in particular, it does not specify the exposure and procedure for using lasers with different wavelengths.

Perhaps these are all studies, unless they don't address particular issues of ALS pathogenesis considered in the context of other diseases.

In vitro studies are poorly informative. Although illumination (λ = 830 nm, PD 10 mW/cm^2^, exposure 5min) decreases nitrite production and modulates the release of IL-10 and γ-interferon by peripheral blood mononuclear cells isolated from disseminated sclerosis patients [44,45], identical patterns in the clinic should not be expected for many reasons, especially if illuminated by multicolored bulbs and “doses” [46].

We have arranged the data presented above in a scheme (Table 1) explaining the possibilities of low-level laser therapy.

## 3. Results

### 3.1. Substantiating LLLT for ALS

Low-level laser therapy should undoubtedly be conducted only in conjunction with already proven supportive treatment options.

Most clinical guidelines from various countries provide the following treatment protocol [7,14].

#### 3.1.1. Drugs

Glutamate releases antagonists and antioxidants.

#### 3.1.2. Breath control

Breath control involves noninvasive pulmonary ventilation, insufflators or exsufflators, nebulizers, and portable aspirators; prevention of respiratory infections by advising on aspiration precautions, adherence to lung hygiene rules, and necessary vaccinations.

#### 3.1.3. Proper nutrition, symptomatic therapy

It is necessary to correct secondary manifestations of the disease: muscle weakness, depression, etc.

In recent years, many published works have confirmed the prospects of LLLT, both for the elimination of primary disorders and prevention of neurodegenerative processes as well as for the elimination of secondary manifestations of relevant diseases [15]. Most often, the authors recommend transcranial illumination, so to speak, in place of disorders. Although this technique is becoming increasingly popular, outstanding issues of optimal (and safe) modes and secondary mechanisms of BA LILI at such localization of exposure remain.

Laser acupuncture has long been used successfully for many diseases, and ALS is no exception [53]. Unfortunately, we couldn't find any appropriate prescriptions.

As noted above, more than 30 years ago, ILBI was recognized as the primary method of LLLT for patients with neurodegenerative diseases [16–19]. However, only recently was ILBI with “classical” parameters (λ = 633 nm, power 10 mW, exposure 15 min) used in ALS to eliminate respiratory dysfunction. A course of LLLT improved breathing, increased peripheral blood oxygen saturation (SpO_2_), and normalized heart rate variability [54]. The authors quite actively refer to the works of Russian authors (which are already good as they know these works), but these authors consider LLLT to be only a symptomatic treatment for eliminating secondary disease manifestations.

This method is quite popular and easy to implement, but unfortunately, many people use ILBI without understanding its essence and or observing its elementary rules. There are numerous cases of using LILI not with the optimal wavelength (638 nm), but in the range of 650–670 nm, where the blood absorbs little laser light, rendering it almost useless [37]. One paper lists a set of meaningless words: “fiber-optic helium-neon diode laser with 5 mW power and 658 nm wavelength.” [55] These are three completely different lasers!

Although physical exercise alone is not productive [56], combining it with laser therapy will undoubtedly be beneficial.

For external exposure and NLBI, it is necessary to use only matrix laser emitting heads containing eight pulsed laser diodes. These are arranged in two rows of four in such a way as to ensure uniform illumination of the area equal to 8 cm^2^ to normalize the power density. According to the known rules, it is necessary to use methods of systemic exposure (LBI, laser acupuncture) and local illumination [57–59].

## 4. Discussion

Thus, the known effects of low-level laser therapy, employed in treating various diseases, and the available, albeit limited, experience of using this method in treating ALS patients allow us to confidently assume the promising prospect of its application in broad clinical practice. However, to obtain a stable result, it is necessary to use the most effective techniques and optimal parameters and to follow the known rules of the procedure.

### 4.1. Particular techniques of LLLT

**Fig. 2:**
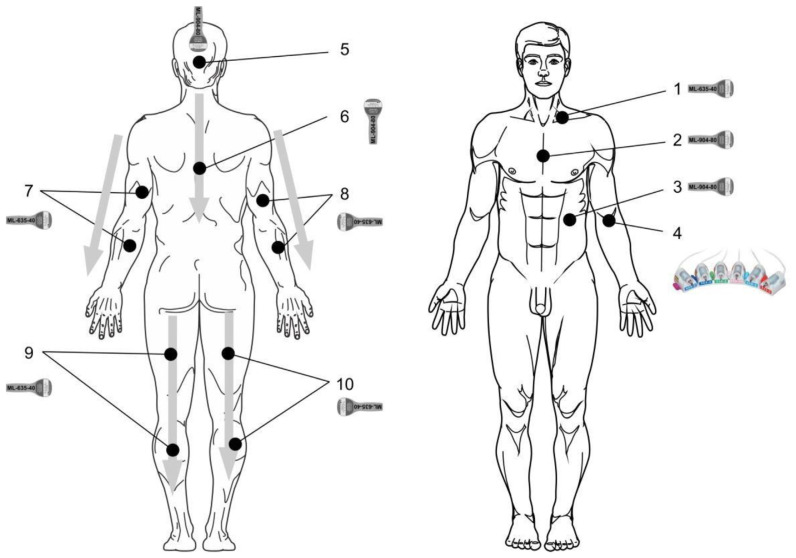
Laser illumination zones in ALS (parameters indicated in the text).

Fig. 2 shows not only the laser illumination zones but also the correct positioning of matrix laser emitting heads.

#### 4.1.1. Laser blood illumination

**WARNING!** Do not perform NLBI (percutaneously) and ILBI (intravenously) procedures on the same day! Excessive maximum exposures are not allowed.

#### 4.1.2. NLBI

Low-level laser therapy devices of Matrix and Lasmik series, matrix pulsed laser-emitting head

(ML-635-40) of the red spectrum: λ = 635 nm, pulsed mode, light pulse duration = 100 ns, frequency = 80 Hz, pulsed power = 30–40 W, exposure = 2 min, stable, zone 1 – supraclavicular area on the left.

#### 4.1.3. Exposure to immunocompetent organs supplements NLBI

Matrix pulsed laser-emitting head (ML-904-80) of the infrared spectrum: λ = 904 nm, pulsed mode, light pulse duration = 100 ns, frequency = 80 Hz, pulsed power = 60–80 W, exposure = 1 min, stable, zones 2 – thymus projection, and 3 – spleen projection.

#### 4.1.4. ILBI-525 technique + laser UV blood illumination (LUVBI®)

KL-ILBI-525-2 laser-emitting head (green spectrum: λ = 525 nm, power at the light guide output = 1.5–2 mW, exposure = 7–10 min) and KLILBI-365-2 laser emitting head (UV spectrum: λ = 365–405 nm, power at the light guide output = 1.5–2 mW, exposure = 3–5 min), with illumination in the median ulnar vein (zone 4 – *v. mediana cubiti*).

The course includes 5–7 daily treatments with alternating spectra (techniques) every other day, for example, **ILBI-525** on Monday, **LUVBI**^®^ on Tuesday, **ILBI-525** on Wednesday, **LUVBI**^®^ on Thursday, and **ILBI-525** on Friday.

#### 4.1.5. Laser acupuncture

KLO-635-5 or KLO-635-15 laser-emitting head with power reduction, with acupuncture nozzle A-3 (λ = 635 nm, continuous or modulated mode, power = 2–3 mW) [60]. To make a prescription, contact a reflexologist.

#### 4.1.6. Local laser illumination

Zone 5 – occiput, transcranial, stable, ML-04-80 infrared matrix-emitting head (λ = 904 nm, pulsed mode, light pulse duration = 100 ns, pulsed power = 60–80 W), located vertically. The frequency varies depending on the procedure:

1–3 treatments – 80 Hz;
4–5 treatments – 1500 Hz;
6–7 treatments – 10,000 Hz;
8–9 treatments – 1500 Hz;
10–12 treatments – 80 Hz.

Zone 6 – along the spine, from top to bottom, from C_1_ to Th_12_, labilely, two times, 30–40 s per pass. Matrix-emitting head (ML-904-80) of the infrared spectrum (λ = 904 nm, pulsed mode, light pulse duration = 100 ns, pulse power = 60–80 W), located vertically. The frequency varies depending on the procedure:

1–3 treatments – 80 Hz;
4–5 treatments – 1500 Hz;
6–7 treatments – 10,000 Hz;
8–9 treatments – 1500 Hz;
10–12 treatments – 80 Hz.

Zones 7–10 – arm and leg muscles, symmetrically (left and right), from top to bottom, labilely, one time, 30–40 s per pass. Matrix pulsed laser-emitting head (ML-635-40) of the red spectrum: λ = 635 nm, pulsed mode, light pulse duration = 100 ns, frequency = 80 Hz, pulsed power = 30–40 W.

## 5. Conclusion

Based on the results of several studies, many years of successful experience with low-level laser therapy in Russia, and our data, this study substantiated the method of laser therapy for ALS and proposed specific LLLT techniques, including two variants of laser blood illumination and local exposure in the affected areas. We consider LLLT as a promising treatment method for ALS but further research is needed.

## Figures and Tables

**Table 1:** Comparison of the abnormalities observed in amyotrophic lateral sclerosis patients and the known BA LILI mechanisms.

Pathology	LILI effect	Literature	Low-level laser therapy method
**Neurodegeneration**	Improving the interaction between astrocytes and neurons	[21,22]	ILBI or NLBI + transcranially
	Protection of motoneurons from the neurotoxic effects of oxygen and nitric oxide reactive species	[23,24]	
	Stimulation of ATP synthesis and energy metabolism in neurons	[25–27]	
	Inflammation reduction	[26,28]	
	Improvement in microcirculation, central blood flow, and cerebral oxygenation	[47–49]	
	Reduction in the size and number of β-amyloid plaques in the neocortex and hippocampus	[50–52]	
**Respiratory distress**	Elimination of distress syndrome and restoration of normal respiration	[34]	ILBI or NLBI + externally to the lung projection, stable
**Muscle atrophy**	Muscle fatigue reduction	[25,27,29–33]	ILBI or NLBI + on muscles, stable or labile
**Muscle stiffness**	Increased muscle performance		
**Muscle weakness, rapid fatigue**	Decreased muscle soreness after physical activity Muscle stiffness reduction		
